# Radiation-free luminal stenting using peroral cholangioscope

**DOI:** 10.1016/j.igie.2025.10.010

**Published:** 2025-10-17

**Authors:** Lin Liu, Hongguang Wang, Liying Tao, Qingmei Guo, Junpeng Liu, Shizhu Liu, Peng Zhan

**Affiliations:** Gastroenterology Department, Jilin People's Hospital, Changchun, Jilin, China

## Abstract

**Background and Aims:**

Traditional endoscopic self-expandable metal stent (SEMS) placement necessitates fluoroscopy guidance when treating gastrointestinal obstruction. This study was performed to report the safety and efficacy of radiation-free peroral cholangioscopy-guided SEMS placement in the treatment of 2 cases with gastrointestinal obstruction.

**Methods:**

This study included 2 patients with gastrointestinal obstruction due to cholangiocarcinoma and sigmoid colon cancer. Peroral cholangioscopy was applied for radiation-free guidewire insertion and SEMS placement.

**Results:**

The guidewire insertion and SEMS placement were successfully accomplished by radiation-free peroral cholangioscopy in both cases. It resulted in significant relief of gastrointestinal obstruction symptoms. In addition, no adverse events such as bleeding or perforation were observed intraoperatively and postoperatively.

**Conclusions:**

Radiation-free peroral cholangioscopy may safely and effectively assist SEMS placement to relieve the symptoms of obstruction caused by gastrointestinal tumors.

## Introduction

Endoscopic self-expandable metal stent (SEMS) placement is a viable approach for addressing gastrointestinal obstructions, such as duodenal obstruction from biliary or pancreatic malignancies and intestinal obstruction from colorectal cancer.^1^ Nonetheless, traditional SEMS placement necessitates fluoroscopic guidance during the procedure to achieve accurate and safe stent positioning. This method, however, may lead to radiologic damage to both the patient and the operating physician.^2^ Furthermore, the blind insertion and passage of the guidewire through the site of obstruction during stent deployment pose risks of bleeding and perforation. Existing literature has reported the use of a 4.9-mm-diameter pediatric nasogastroscope to visually guide the guidewire through colorectal obstructions, ensuring safer stent positioning.^3^ In this study, we present 2 cases of SEMS placement using a thinner and more-flexible peroral cholangioscope without the use of fluoroscopy, effectively managing malignant gastrointestinal obstruction.

## Methods

### Endoscopic technique

The 2 cases presented in this article were successfully managed using the innovative “scope-in-scope” technique, in which a peroral cholangioscope (CDS22001, eyeMAX; Micro-Tech, Nanjing, China) was inserted through the biopsy channel of a colonoscope (CF-HQ290I; Olympus, Tokyo, Japan). The colonoscope was advanced to the vicinity of the tumor-induced stricture, and the peroral cholangioscope was introduced via its working channel. Under direct visualization, the peroral cholangioscope allowed precise identification of the opening to the stricture and successful advancement through it. The length of the malignant stricture was precisely assessed using the graduated markings on the peroral cholangioscope shaft. Passage through the stricture was confirmed via peroral cholangioscopy by visualizing the dilated intestinal lumen and normal mucosa in the colon and duodenum beyond the stricture. A guidewire (VDK-ZGW-88-450-A; Vedkang, Changzhou, China) was then advanced through the instrument channel of the peroral cholangioscope, carefully passing beyond the stricture. In the colonic case, the guidewire was positioned 15 cm upstream from the tumor, whereas in the duodenal case, it was placed 10 cm beyond the distal end of the tumor. Maintaining the guidewire in a fixed position, the peroral cholangioscope was slowly withdrawn. During withdrawal, the position and axial alignment of the guidewire with the intestinal tract were reconfirmed to ensure that the stent could be accurately deployed in a parallel manner within the stricture. Additionally, the stenosis length was re-evaluated using the peroral choledochoscope's shaft markings to select a stent of appropriate length. Under combined guidance from the guidewire and endoscopic visualization, a metallic stent (NST64-331-26.100; Micro-Tech) was advanced across the tumor site, then gradually deployed, and fully released. The stent was selected to extend 2 cm beyond the end of the tumor. During the deployment, the insertion depth of the stent delivery system could be dynamically adjusted to precisely control the stent's position relative to the endoscopically visible edge of the stenosis. After stent placement, the peroral cholangioscope was reintroduced through the stent lumen to confirm that the stent adequately covered both ends of the stricture. Finally, an upright abdominal plain radiograph was obtained 48 hours postoperatively to confirm the final positioning of the stent. The following outlined the procedural details of 2 clinical cases.

## Results

**Figure 1:**
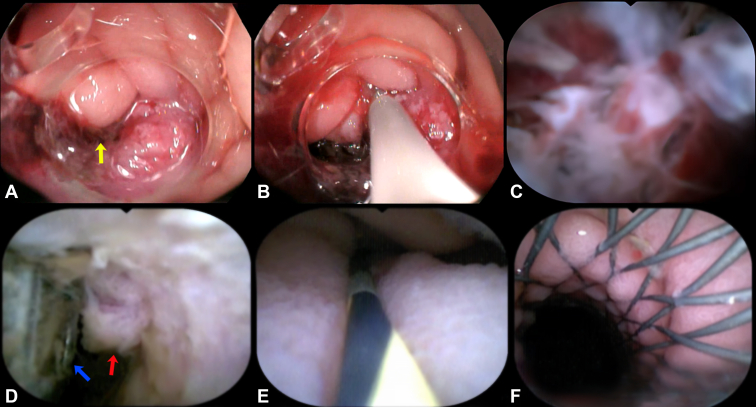
Radiation-free peroral cholangioscopy-guided self-expandable metal stent placement for cholangiocarcinoma with duodenal obstruction. **A,** Gastroscopy revealed severe duodenal bulb stenosis (*yellow arrow*). **B,** Peroral cholangioscope (white catheter) identified the opening in the stenosis and was advanced through this. **C,** Peroral cholangioscope directly observed the stenotic area. **D,** Peroral cholangioscope identified a metal stent (*blue arrow*) at the duodenal papilla and a mass (*red arrow*) opposite. **E,** The guidewire passed through the stenotic segment to the proximal jejunum beyond the obstruction. **F,** Enteral stent deployed across the duodenal stricture.

**Case 1:** A 67-year-old male patient underwent placement of a biliary metal stent for bile duct cancer 1 year ago. The patient presented at the hospital 1 week earlier with intermittent, vague upper abdominal pain, accompanied by nausea, vomiting, and black stools. Laboratory tests indicated mild anemia, hypokalemia, and a positive fecal occult blood test. Gastroscopy showed severe stenosis of the duodenal bulb and an irregular mass with bleeding, which obstructed the passage of the gastroscope (Fig. 1A). Using the peroral cholangioscope (eyeMAX), we located the narrowed gap, and insertion was performed under direct visualization (Fig. 1B). During endoscopic insertion with the peroral cholangioscope, significant amounts of necrotic tissue accompanied by blood clots and irregularly shaped villous elevations were observed in the duodenum, leading to marked stenosis of the duodenal lumen (Fig. 1C). The peroral cholangioscope advanced through the stenosis into the descending duodenum, revealing a metal stent at the duodenal papilla encrusted with food debris (Fig. 1D, *blue arrow*) and an irregular mass causing eccentric stenosis on the opposite side (Fig. 1D, *red arrow*). A guidewire was inserted using the peroral cholangioscope, navigating through the stenosis into the proximal portion of the jejunum as far as possible (Fig. 1E). Under guidewire guidance, a metal uncovered stent (26-mm diameter, 100-cm length; Micro-Tech) was positioned at the site of duodenal obstruction (Fig. 1F). Subsequently, the peroral cholangioscope successfully traversed the metal stent and advanced into the distal duodenum (Video 1, available online at www.igiejournal.org).

**Figure 2:**
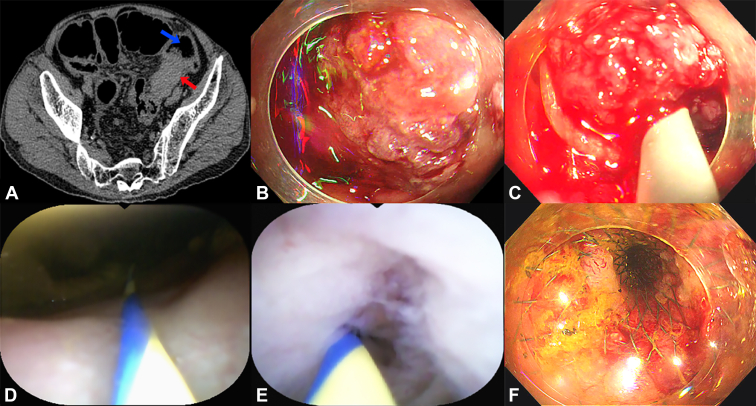
The imaging examination and radiation-free peroral cholangioscopy-guided self-expandable metal stent placement for sigmoid colon cancer with intestinal obstruction. **A,** Computed tomography examination suggested a sigmoid colon tumor (*red arrow*) causing secondary intestinal obstruction (*blue arrow*, dilated colon proximal to the tumor). **B,** Colonoscopy showed a very friable sigmoid tumor mass causing severe colonic stricture. **C,** The peroral cholangioscope (white catheter) identified the narrowing in the stricture and was advanced through this area. **D,** The guidewire crossed the stenosis to reach the proximal dilated bowel lumen. **E,** Observed tumor stenosis during peroral choledochoscope withdrawal. **F,** Colonic stent placed across sigmoid stricture.

**Case 2:** A 73-year-old male patient exhibited symptoms of cessation of bowel movements, abdominal distension, pain, nausea, and vomiting for 10 days, accompanied by elevated levels of hypersensitive C-reactive protein, procalcitonin, and hypoproteinemia on laboratory test results. Computed tomography scan revealed a suspected cancerous mass in the sigmoid colon leading to invasion of the serosa layer and intestinal obstruction, along with fecal stone formation, enlarged peripheral lymph nodes, and pelvic effusion (Fig. 2A). To alleviate colonic obstruction and facilitate subsequent elective surgery, the patient underwent sigmoid stent placement. Colonoscopy revealed an irregular nodular circumferential space-occupying lesion in the sigmoid colon, with spontaneous bleeding on the surface. The texture of the lesion tissue was fragile and prone to bleeding on contact (Fig. 2B). The stenosis was identified and successfully inserted under direct visualization using the peroral cholangioscope (eyeMAX) (Fig. 2C). The peroral cholangioscope traversed the tumor stenosis directly to the dilated colon upstream from the stricture, and then the guidewire was inserted under direct visualization. The guidewire was successfully navigated across the stenotic region and accurately positioned within the proximal intestinal segment (Fig. 2D). Withdrawal examination identified irregular villous elevation of the mucosa at the severe stricture caused by the tumor, with the guidewire positioned at the center of the stricture (Fig. 2E). Afterward, a metal uncovered stent (26-mm diameter, 100-cm length; Micro-Tech) was placed under the guidance of the guidewire, with visible fecal drainage (Fig. 2F, Video 2, available online at www.igiejournal.org). The patient underwent radical transabdominal surgery for sigmoid colon cancer 2 weeks after intestinal stent placement, and postoperative pathology indicated adenocarcinoma.

**Figure 3:**
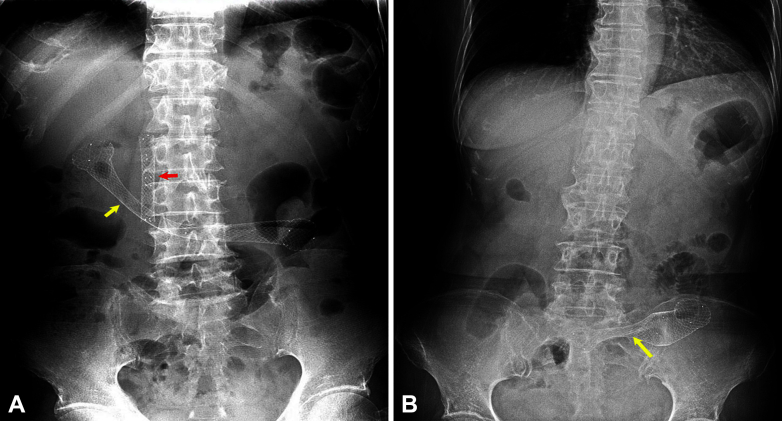
The upright abdominal plain-film examination performed 48 hours after the placement of the self-expandable metal stent in cases of gastrointestinal obstruction. **A,** The duodenal metallic stent (*yellow arrow*) was accurately positioned and demonstrated excellent expansion. Additionally, the previously implanted biliary metallic stent (*red arrow*) remained visible. **B,** The colonic stent (*yellow arrow*) was accurately positioned and well expanded.

Radiation-free peroral cholangioscopy-assisted SEMS placement took 27 minutes in the colon and 33 minutes in the duodenum. Gastrointestinal obstruction symptoms in both patients were markedly relieved following SEMS placement, and a liquid diet was resumed within 6 hours postoperatively. The position of the stent was confirmed by an upright abdominal plain radiograph 48 hours after the operation (Fig. 3). No adverse events, including bleeding or perforation, were observed intraoperatively or postoperatively.

## Discussion

Cholangiocarcinoma, gastric cancer, and metastases to the duodenum or proximal jejunum can lead to duodenal obstruction. Colorectal cancer is prevalent worldwide, with 10% to 28% of patients experiencing acute obstruction symptoms.^1^ Endoscopic SEMS placement is a commonly used clinical intervention for alleviating intestinal obstruction, aiming to enhance quality of life.^4^ Traditionally, gastrointestinal SEMS placement involves fluoroscopy or combined endoscopic-fluoroscopy guidance. However, fluoroscopy-guided procedures expose patients and providers to radiation with its risks. The blind insertion of the guidewire through the obstruction often necessitates fluoroscopy assistance. In severe cases, excessive manipulation of the guidewire can elevate the risks of perforation^5^ and bleeding. De Gregorio et al^6^ found an average radiation dose of 3378 dGycm^2^ in 467 fluoroscopy-guided colon stent placements, underscoring the potential harm from fluoroscopy exposure. In addition, the success rate of endoscopic colonic SEMS placement is suboptimal, with a failure rate ranging from 2% to 10%.^7^ In a cohort of 64 colorectal cancer patients, the use of a pediatric nasogastroscope to guide metal stent placement reduced both immediate and delayed adverse events.^8^

The peroral cholangioscope is available in various models, with the 9F model specifically chosen for this study because of its thinner diameter compared with that of the transnasal gastroscope. This model allows for flexible maneuvering and provides clear visualization. In our 2 cases, there was potential for perforation and bleeding from blind guidewire insertion through severe duodenal and colonic obstructions. The peroral cholangioscope is equipped with suction and water-filling channels, facilitating fluid injection and aspiration to navigate obstructions, thereby minimizing aspiration risk. The peroral cholangioscope with direct visualization enabled accurate identification and advancement through the strictures without the need for fluoroscopy. After stent placement, it evaluates stent coverage and patency directly. Its direct visualization capability ensures accurate guidewire and stent placement, avoiding fluoroscopy exposure. No instances of bleeding or perforation were observed during or after placement. Following the procedure, obstructive symptoms ameliorated, allowing the patients to resume oral intake on the same day.

In conclusion, peroral cholangioscopy serves as a potentially valuable technique for the precise and safe placement of SEMSs to bypass intestinal obstructions without the need for fluoroscopy. Additional larger studies are needed to assess this technique further.

## Patient Consent

The patients in this article have given written informed consent to publication of their case details.

## Disclosure

All authors disclosed no financial relationships.

## Supplementary data

Video 1 Radiation-free peroral cholangioscopy-guided placement of self-expandable metal stents for cholangiocarcinoma associated with duodenal obstruction.

Video 2 Radiation-free peroral cholangioscopy-guided placement of self-expandable metal stents for sigmoid colon cancer associated with intestinal obstruction.
